# Advanced multiple sclerosis: an exploratory study on a neglected patient population

**DOI:** 10.1007/s00415-025-13296-6

**Published:** 2025-08-03

**Authors:** Omar Keritam, Oliver Ascoli, Andrea Harsanyi, Hakan Cetin, Thomas Berger, Matthias Unseld, Paulus Stefan Rommer

**Affiliations:** 1 Department of Neurology, Medical University of Vienna, Waehringer Guertel 18-20, 1090 Vienna, Austria; 2 Comprehensive Center for Clinical Neurosciences and Mental Health, Medical University of Vienna, Vienna, Austria; 3 Haus Der Barmherzigkeit Seeböckgasse, Vienna, Austria

**Keywords:** Advanced multiple sclerosis, Palliative care, Co-morbidities, Polypharmacy

## Abstract

**Background:**

Multiple sclerosis (MS) is a chronic immune-mediated disease that can cause severe physical and cognitive disability. While modern therapies have improved outcomes in relapsing MS, patients with advanced disease remain underserved. In this stage, neurodegeneration dominates, treatment options are limited, and care becomes complex. Yet individuals with advanced MS are largely absent from trials, registries, and structured care pathways, leaving a major evidence gap.

**Objectives:**

To characterize the clinical, social, and treatment-related profile of patients with advanced MS in institutional care.

**Methods:**

We conducted an exploratory, retrospective study of patients with advanced MS (EDSS ≥ 6.5) admitted to a long-term care facility in Vienna. Data on disease history, comorbidities, medications, cognitive and functional status, and social background were extracted from medical records.

**Results:**

Thirty-four patients were included (73.5% female; median age at admission: 54.1 years). Most had secondary progressive MS (85.3%). Disease-modifying therapy (DMT) exposure was limited; only one patient received DMT during care. Comorbidity and polypharmacy were frequent. EDSS progression occurred in 50%. The Braden Scale was the only score differing significantly between cohorts.

**Conclusions:**

This study highlights care gaps in advanced MS and the need for tailored strategies in institutional care settings.

## Introduction

Multiple sclerosis (MS) is a chronic immune-mediated disease of the central nervous system (CNS), characterised by episodes of inflammation and by progressive neurodegeneration [1]. In most cases MS starts with a relapsing–remitting course. Over time, however, many patients develop disease progression, leading to accumulation of neurological and physical disability [2].

Since the early 1990s the therapeutic landscape in MS has changed substantially with the advent of numerous disease-modifying therapies (DMTs) targeting inflammatory processes [3]. These treatments have tremendously improved outcomes for patients with relapsing MS, reducing relapse rates and delaying conversion to secondary progression [3–5]. However, for individuals with primary or secondary progressive MS (PPMS or SPMS), especially in later stages where neurodegeneration predominates and inflammation subsides, therapeutic options remain limited [6–10]. Patients with advanced MS, defined as an Expanded Disability Status Scale (EDSS) score of ≥ 6.5 [11], often experience profound physical and cognitive impairments, high levels of dependency, and a substantial psychological and social burden [12, 13]. Consequently, symptomatic therapies and long-term nursing support become the mainstay of care management [14, 15]. This shift has serious implications: patients with high disability scores (e.g., EDSS ≥ 6.5) incur substantially greater healthcare costs, require intensive caregiving, and frequently experience diminished quality of life [16]. Treatment of those patients is frequently complicated by multimorbidity, psychiatric comorbidities, polypharmacy, and the breakdown of family and social support structures [17–19].

Despite their clinical and economic significance, patients with advanced MS remain severely underrepresented in research. They are commonly excluded from clinical trials either due to mobility impairments or comorbidities, or due to their age [20]. Even large-scale national MS datasets rarely provide granular insight into patients in the highest disability strata [21]. This has created a persistent evidence gap regarding the clinical course, care needs, and treatment patterns of individuals with long-standing progressive MS. Furthermore, few studies explore the lived experiences, social circumstances, or historical treatment trajectories of these patients – particularly those requiring institutional care.

In this exploratory, retrospective cohort study, we aimed to address this knowledge gap by providing a detailed clinical and demographic characterization of patients with advanced MS (EDSS ≥ 6.5) receiving long-term care at a specialized palliative care facility. We analyzed disease history, comorbidities, prior treatments, and social context to better understand disease progression and care dynamics in this otherwise neglected population. Our findings are intended to inform future research, highlight unmet needs, and guide the development of tailored care strategies for patients with advanced MS.

## Methods

### Study design and participants

This explorative study included 34 adult patients with advanced MS, who were in need of constant care and who were admitted to the medical care facility “Haus der Barmherzigkeit Seeböckgasse” (HdB) between January 2014 and September 2024. All patients had an Expanded Disability Status Scale (EDSS) ≥ 6.5 at the time of admission, which was defined as severe MS according to the European Academy of Neurology guidelines on palliative care in MS [11]. Data on social and family history, as well as clinical information, were extracted from hospital records between 2005 and September 2024 in the databases of the HdB and the Department of Neurology at the Medical University of Vienna. Where possible, a questionnaire was used to collect additional historical information. Informed consent forms were obtained from all patients. This study was approved by the ethics committee of the Medical University of Vienna (EK No. 1117/2024).

### Statistical analysis

Descriptive statistics for demographic and clinical parameters were summarized using medians and interquartile ranges. EDSS values at three time points — prior to admission (T1), at admission (T2), and at last follow-up (T3) — were compared using the Kruskal–Wallis test with Dunn’s post-hoc test. The change between T2 and T3 was further assessed using the Wilcoxon matched-pairs signed-rank test. Exploratory analyses were conducted to evaluate associations between selected clinical, demographic, and social factors and disease progression.

The time difference between MS diagnosis and admission to HdB (Δt_prog_) was used as an estimate of disease progression prior to reaching “advanced” MS. Results from a magnetic resonance imaging (MRI) of the brain performed before admission were extracted, and Δt_MRI_ was defined as the time interval between the date of the MRI result and the date of admission. The patient cohort was grouped according to the following parameters for exploratory subgroup analyses: gender (male vs. female), main prior occupation (office vs. other), level of education (primary school/apprenticeship vs. high school/university), marital status after diagnosis (married vs. divorced/widowed), lifetime smoking (yes vs. no), cardiovascular disease (yes vs. no), psychiatric disorder (yes vs. no), and brain atrophy on MRI (yes vs. no). Δt_prog_ was compared between subgroups using the Mann–Whitney test, except for the last variable, for which Δt_MRI_ was used. Associations between Δt_prog_ and Mini-Mental Status Examination (MMSE, 30-point test used to screen for cognitive impairment and assess mental status), Braden Scale (23-point scale used to assess risk of developing pressure ulcers by evaluating sensory perception, skin moisture, physical activity, mobility, nutritional status, and friction/shear), as well as Barthel Index (100-score index used to measure a person’s ability to perform basic activities of daily living (ADLs)) at admission were analysed using Pearson’s correlation.

For exploratory analysis of disease progression after admission, patients were divided into two groups based on EDSS progression (worsening by ≥ 0.5 points vs. no worsening) between T2 and T3. EDSS, MMSE, Braden Scale and Barthel Index at T2 were compared using the Mann–Whitney test. Fisher’s exact test was used to compare the frequencies of the following dichotomous parameters: gender (male vs female), main prior occupation (office vs other), level of education (primary school/apprenticeship vs high school/university), marital status after admission (married vs divorced/widowed), lifetime smoking (yes vs no), cardiovascular disease (yes vs no), psychiatric disorder (yes vs no).

A two-sided p-value < 0.05 was considered statistically significant; however, statements on statistical significance should be interpreted as descriptive only. Statistical analysis was performed using GraphPad Prism version 10.4.1 for macOS (GraphPad Software, Boston, Massachusetts, USA).

## Results

Within the total cohort, 85.3% of patients had a diagnosis of SMPS at the timepoint of admission, while 8.8% had PPMS. The MS subtype was unknown in the remaining 5.9%. Females accounted for 73.5% of the cohort. The median age at MS diagnosis was 32.0 years (IQR 26.0–43.5), and the median age at admission to HdB was 54.1 years (IQR 48.6–69.9). The median time from diagnosis to admission was 20.3 years (IQR 13.2–31.6). By the time of data extraction, 14 patients had died. None of the patients experienced a relapse during their stay at HdB (Table 1).

**Table 1:** Clinical data

	n = 34
Type of MS	
SPMS	29 (85.3%)
PPMS	3 (8.8%)
Unknown	2 (5.9%)
Female	25 (73.5%)
Median age at diagnosis of MS in years (IQR)	32.0 (26.0–43.5)
Unknown	1 (2.9%)
Median age at admission to HdB in years (IQR)	54.1 (48.6–69.9)
Median time diagnosis to admission into HdB in years (IQR)	20.3 (13.2–31.6)
Unknown	1 (2.9%)
EDSS	
Median last EDSS before admission (IQR)	6.8 (6.1–7.5)
Median age at last EDSS before admission in years (IQR)	52.8 (43.3–66.3)
Unknown	18 (52.9%)
Median EDSS at admission (IQR)	8.5 (7.5–9.0)
Unknown	2 (5.9%)
Median last documented EDSS	9.0 (8.5–9.5)
Median age at last documented EDSS in years (IQR)	65.8 (58.3–76.1)
Other scores at admission	
Median MMSE (IQR)	24.0 (16.5–28.0)
Unknown	9 (26.5%)
Median Barthel Index (IQR)	22.5 (5.0–43.8)
Unknown	14 (41.2%)
Median Braden Scale (IQR)	14.5 (12.0–17.5)
Unknown	0 (0.0)
Co-morbidities	
Other neurological disease	24 (70.6%)
Cardiovascular	21 (61.8%)
(Past) malignancy	7 (20.6%)
Other autoimmune disease	1 (2.9%)
Psychiatric disorders	
Number of patients with psychiatric diagnosis	27 (79.4%)
Psychoorganic syndrome	16 (47.1%)
Dementia or mild cognitive impairment	10 (29.4%)
Depression	9 (26.5%)
Psychosis	3 (8.8%)
Anxiety disorder	2 (5.9%)
Personality or behavioral disorder	2 (5.9%)
Unknown	1 (2.9%)
Number of deceased patients	14 (41.2%)
Median age at death in years (IQR)	75.3 (57.7–83.6)
Cause of death	
Cardiac failure	13 (92.9%)
Pulmonary embolism	1 (7.1%)

*EDSS* Expanded Disability Status Scale, *HdB* “Haus der Barmherzigkeit” (name of long-term care facility in Vienna), *IQR* interquartile range, *MMSE* Mini-Mental Status Examination. *MS* multiple sclerosis, *PPMS* primary-progressive multiple sclerosis. *SPMS* secondary-progressive multiple sclerosis

**Fig. 1:**
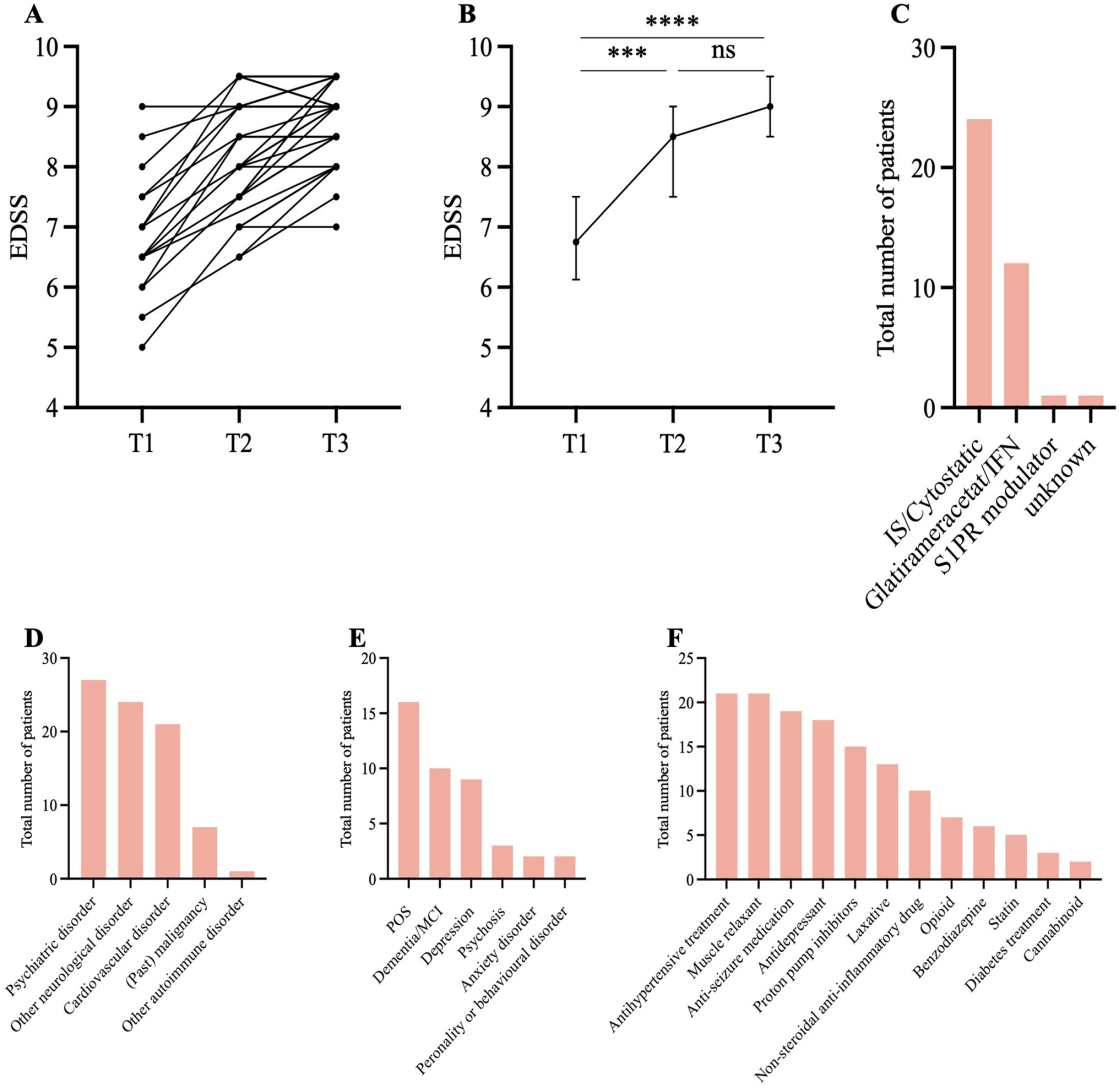
**A**,** B** On a group level, EDSS progressed only between T1 and T2, but not between T2 and T3. However, post-hoc analysis with the Wilcoxon matched pairs signed rank test revealed a significant difference between T2 and T3. 17 patients experienced worsening after admission to HdB. **C** Only one patient received a DMT after admission. During their disease course, however, 24 patients received cytostatic or immunosuppressive treatment, while 12 received glatiramer-acetate or interferon (IFN). sphingosine-1-phosphate receptor (S1PR) modulator was prescribed to only one patient. **D, E** The most common comorbidities were psychiatric disorders (e.g. psychoorganic syndrome, POS, dementia, or mild cognitive impairment, MCI), other neurological syndromes (e.g. seizures), and cardiovascular disease (e.g. arterial hypertension). **F** Most frequently prescribed medications were antihypertensive medication (n = 21), anti-seizure medication (n = 19) or antidepressants (n = 18). Additional co-medications reflect symptoms commonly associated with MS and requiring treatment. 21 patients received muscle relaxants, 13 patients received laxatives, and 7 patients received opioids

Only one patient received a DMT (glatiramer-acetate) during the stay at HdB. In the past, 12 patients (35.3%) had received glatiramer-acetate or interferons (IFN), and 24 (70.6%) had been treated with immunosuppressive medication, such as cortisone, azathioprine, mitoxantrone, or cyclophosphamide. One patient had been treated earlier with a sphingosine-1-phosphate receptor modulator. The most common comorbidities were psychiatric disorders (79.4%), followed by other neurological disorders (70.6%), cardiovascular disorders (61.8%), past malignancies (20.6%), and other autoimmune disorders (2.9%). Neurological co-morbidities included seizures, past cerebral infarction, trigeminal neuralgia, or diabetic neuropathy. On average, patients were prescribed seven concomitant medications. The most commonly used were antihypertensives or muscle relaxants (61%), anti-seizure medications (55.9%), antidepressants (52.9%), proton pump inhibitors (44.1%), and laxatives (38.2%). Additional medications included non-steroidal anti-inflammatory drugs (29.4%), opioids (20.6%), benzodiazepines (17.6%), statins (14.7%), antidiabetic agents (8.8%), and cannabinoids (5.9%). Lifetime smoking was reported in 52.9% of the cohort (Table 2 and Fig. 1).

**Table 2:** MS treatment and co-medication

	n = 34
MS treatment	
Past MS treatment (all patients)	
Glatirameracetat/interferon	12 (35.3%)
Sphingosine-1-phosphate receptor modulator	1 (2.9%)
Immunosuppressive/cytostatic treatment	24 (70.6%)
Unknown	1 (2.9%)
At time of data extraction (deceased patients excluded)	
Copaxone	1 (0.05%)
Co-medication	
Median number of prescribed drugs (IQR)	7.0 (5.8–10.3)
Antihypertensive treatment	21 (61.8%)
Muscle relaxant	21 (61.8%)
Anti-seizure medication	19 (55.9%)
Antidepressant	18 (52.9%)
Proton pump inhibitors	15 (44.1%)
Laxative	13 (38.2%)
Non-steroidal anti-inflammatory drug	10 (29.4%)
Opioid	7 (20.6%)
Benzodiazepine or analogue	6 (17.6%)
Statin	5 (14.7%)
Diabetes treatment	3 (8.8%)
Cannabinoid	2 (5.9%)

*IQR* interquartile range, *MS* multiple sclerosis

Most patients grew up in an urban environment (70.6%), whereas 17.6% in rural areas. A small proportion (11.8%) were born and raised abroad. A positive family history of MS was rare, with only one patient reporting an affected relative, while 23 patients stated a negative family history, and in 10 patients this was unknown. 20.6% of patients had completed only primary school, 38.2% had training in a skilled trade, 20.6% graduated from high school, and 11.8% held a university degree. The majority of patients (61.8%) had worked in office-based professions. The median time from diagnosis to premature retirement due to loss of work capacity was 3.0 years (IQR 1.0–13.0). Marital status evolved over the disease course: prior to diagnosis, 58.8% of patients were married and one patient was divorced. After diagnosis, 50.0% remained married, 26.4% were divorced, and one patient was widowed. After admission to HdB, only 9 patients (26.4%) were still married, whereas 14 patients (41.2%) were divorced, and 4 patients were widowed (Tables 3 and 4).

**Table 3:** Social and family history

	n = 34
Childhood residency	
Urban	24 (70.6%)
Rural	6 (17.6%)
Born abroad	4 (11.8%)
Family history of MS — positive	1 (2.9%)
Unknown	10 (29.4%)
Highest education	
Primary school	7 (20.6%)
Vocational/apprenticeship	13 (38.2%)
High school	7 (20.6%)
University degree	4 (11.8%)
Unknown	3 (8.8%)
Main occupation	
Office-based	21 (61.8%)
Other	12 (35.3%)
Unknown	1 (2.9%)
Time to work incapacity in years (IQR)	3.0 (1.0–13.0)
Unknown	19 (55.9%)

*IQR* interquartile rangem, *MS* multiple sclerosis

**Table 4:** Marital status prior to diagnosis of MS, after diagnosis of MS and after admission to HdB

Disease stage	Never married	married	divorced	widowed	unknown	n (total)
Prior to MS diagnosis	12 (35.3%)	20 (58.8%)	1 (2.9%)	0 (0.0%)	1 (2.9%)	34
Following MS diagnosis	6 (17.6%)	17 (50.0%)	9 (26.4%)	1 (2.9%)	1 (2.9%)	34
After admission to HdB	6 (17.6%)	9 (26.4%)	14 (41.2%)	4 (11.8%)	1 (2.9%)	34

*HdB* “Haus der Barmherzigkeit” (name of long-term care facility in Vienna), *MS* multiple sclerosis

EDSS scores showed significant progression on a group level between T1 (median 6.8, IQR 6.1–7.5) and T2 (median 8.5, IQR 7.5–9.0, p = 0.0009), but not between T2 and T3 (median 9.0, IQR 8.5–9.5; p = 0.1436). However, a post-hoc Wilcoxon matched-pairs test revealed a significant difference between the EDSS at T2 and T3 (median difference 0.5, IQR 0.0–1.0; p = 0.0004). Disease progression (defined as an EDSS increase by ≥ 0.5) was observed in 17 patients (50%), while 15 patients (44.1%) remained stable. The median time between T2 and T3 was 6.0 years (IQR 4.0–11.8). Brain MRIs, performed a median of 9.0 years prior to admission (IQR 2.7–17.1), revealed cerebral atrophy in 20.6% of patients. Cognitive and functional assessments at admission showed a median MMSE score of 24.0 (IQR 16.5–28.0), a median Barthel Index of 22.5 (IQR 5.0–43.8), and a median Braden Scale score of 14.5 (IQR 12.0–17.5) (Table 1 and Fig. 1).

An exploratory analysis revealed no statistically significant associations between Δt_prog_ and key demographic, social, or clinical factors. Progression times did not differ significantly between men and women, between patients with office-based versus manual occupations, or between different educational backgrounds. Similarly, marital status, smoking history, and the presence of psychiatric or cardiovascular comorbidities showed no significant influence on disease progression. Neuroimaging findings, including the presence of brain atrophy, were also not associated with differences in Δt_MRI._ Additionally, no correlations were found between progression time and functional or cognitive assessments, including MMSE, Barthel Index, or Braden Scale. At T2, there were no significant differences in EDSS, MMSE or Barthel Index between patients who later experienced EDSS progression (≥ 0.5 points) and those who remained stable. Likewise, no significant differences were observed in sex, occupation, education level, marital status, smoking history, cardiovascular disease or psychiatric comorbidities. The only significant difference was found in the Braden Scale at T2, which was lower in stable patients (n = 15, median 13.0, IQR 11.0–15.0) compared to those with subsequent EDSS progression (median 16.0, IQR 13.5–20.5, p = 0.0047).

## Discussion

In this single-center cohort study, we provide a comprehensive clinical, demographic, and historical description of patients with advanced MS (EDSS ≥ 6.5) receiving long-term care in a specialized palliative care center. Our findings offer insight into a severely disabled and understudied—neglected—patient population.

Only one patient received a DMT during the stay at HdB, and overall, lifetime DMT exposure was scarce in this cohort. This suggests that our findings may reflect to a certain extent the natural course of advanced MS in the absence of effective DMTs. EDSS progression was observed in 50.0%, most likely driven by ongoing neurodegenerative mechanisms. In light of current practice, where high-efficacy DMTs are available early and often continued into SPMS, our cohort likely represents a historical extreme. Nonetheless, the structural and caregiving needs described here remain highly relevant. However, contribution from comorbidities, aging, or complications of immobility cannot be excluded. Treatment in this cohort primarily focused on symptomatic and palliative care, which is also reflected in the lack of systematically gathered follow-up biomarkers. Cerebral MRI after admission, for example, was only utilized in rare instances, e.g., for the monitoring of disease activity in the patient who underwent treatment with glatiramer-acetate.

The relatively young median age at admission (54 years) paired with a high EDSS (8.5) highlights the severity of disability in this population. Compared to data from the German MS registry, where the mean EDSS was 4.2 in patients aged 55–64 years, and 5.3 in those over 65 years [21], our cohort appears substantially more disabled. This contrast most likely resulted from a selection bias which is rooted in the single center design of our study, but may also relate to differences in prior treatment exposure, as over half of the patients in the German registry still received DMTs beyond the age of 55. Contemporary DMTs were not available in the early disease stages of most of our patients and most likely were not prescribed due to lack of inflammatory activity in later disease stages. However, the growing body of evidence suggests that continuous or even delayed DMT use may decelerate disability progression even in later disease stages [22]. Additionally, discontinuation of DMTs may result in faster disability accumulation in progressive disease forms [22, 23]. Therefore, specific DMTs, like ocrelizumab, IFN-beta and siponimod [24], may become imperative in the treatment of progressive MS.

Exploratory subgroup analysis did not identify any demographic, social, or clinical factors that significantly associated with the time to advanced MS. Notably, earlier admission was more frequently observed in patients divorced or widowed after diagnosis, possibly reflecting the role of psychosocial strain or lack of social support, although that finding was not statistically significant. These results align with prior work linking social support to disease coping and quality of life in MS [25].

Interestingly, EDSS, MMSE, and Barthel Index at T2 did not significantly differ between patients who further progressed and those who remained stable. However, the Braden Scale was higher in the progression group, possibly reflecting a better functional status at baseline. The Braden Scale assesses the risk for pressure ulcers and includes parameters such as mobility, activity level, sensory perception, and nutrition. Interestingly, higher Braden scores at admission were associated with subsequent EDSS progression. While this appears counterintuitive, it might reflect a better functional status at baseline, making progression more detectable. Alternatively, the association may be incidental and should be interpreted with caution, as the prognostic value of the Braden Scale in MS is unknown. Given the exploratory design and limited sample size, no conclusions regarding causality can be drawn.

Several limitations must be acknowledged. The retrospective nature and reliance on medical records may have introduced information and selection bias, reflecting inherent limitations of such study designs. Furthermore, extracted time points of MRI or EDSS at T1 varied significantly among our patient cohort and did not reflect disease status in the time period close to admission. Consequently, various statistical analyses should be interpreted with caution. Second, the single center design of our study could restrict replicability in other medical facilities with varying care models, especially across different countries. Third, the small sample size limits statistical power and generalizability to a broader patient cohort, particularly for specific subgroups. Fourth, the absence of a control group of similarly disabled patients with prior long-term DMT exposure precludes conclusions regarding treatment effects in advanced MS. However, as most patients reached advanced stages before high-efficacy therapies became available, our cohort likely reflects the natural history of advanced MS without modern immunotherapy. Lastly, longitudinal biomarker data (e.g., MRI) were mostly limited to pre-admission periods, and no serological biomarkers (e.g., neurofilaments) were collected during institutional care, restricting insight into ongoing disease activity. In summary, utilizing a multicenter, prospective and longitudinal case–control design with a significantly higher patient number would resolve most of these limitations.

Despite these limitations, the study provides novel insights into a population that remains largely invisible in clinical research, routine care, registries, and guidelines. Patients with advanced MS bear the highest disease burden, yet are frequently excluded from the evidence base that informs treatment strategies. Our data illustrate that, once patients transition to institutional care, they often receive little or no disease-modifying treatment — and even symptomatic management is frequently limited — despite considerable residual life expectancy and ongoing progression. This underscores substantial gaps in the care of patients with advanced MS, including their underrepresentation in clinical research and registries, the limited use of disease-modifying therapies, the absence of structured multidisciplinary care pathways, inadequate monitoring of disease activity, and insufficient access to symptomatic treatments and psychosocial support. Future efforts should prioritize tailored strategies to address these gaps, preserve function, prevent complications, and maintain quality of life in this underserved population. Registries could facilitate our understanding of changes in the natural disease course and the pathophysiology of advanced MS, especially in light of the growing landscape of DMTs. As clinical focus increasingly shifts toward preserving function, preventing complications, and maintaining quality of life in later disease stages, access to specialized MS care and individualized management and treatment approaches, including DMTs as well as supportive or symptomatic treatment options, become crucial. Importantly, Infrastructures that support psychosocial and socioeconomic needs—such as social counselling, peer support, psychological care, and caregiver education—should be expanded to ensure truly patient-centered, comprehensive management in advanced MS.

## Conclusion

This study highlights the profound unmet needs and disease burden in patients with advanced MS. Future care strategies should address both individualized medical management and psychosocial and functional support to preserve quality of life.

## Data Availability

No patient data or study related documents are shared within this paper. Reasonable requests from qualified investigators will be considered by the corresponding author in accordance with applicable privacy regulations.

## Declarations

### Additional information

Oliver Ascoli collected the data for his diploma thesis as part of this project.
